# Psychological and biological resilience modulates the effects of stress on epigenetic aging

**DOI:** 10.1038/s41398-021-01735-7

**Published:** 2021-11-27

**Authors:** Zachary M. Harvanek, Nia Fogelman, Ke Xu, Rajita Sinha

**Affiliations:** 1 Department of Psychiatry, Yale University, New Haven, CT USA; 2 Yale Stress Center, Yale University, New Haven, CT USA; 3 Department of Psychiatry, Connecticut Veteran Healthcare System, West Haven, CT USA; 4 Department of Neuroscience, Yale University, New Haven, CT USA; 5 Child Study Center, Yale University, New Haven, CT USA

**Keywords:** Psychology, Biomarkers, Physiology

## Abstract

Our society is experiencing more stress than ever before, leading to both negative psychiatric and physical outcomes. Chronic stress is linked to negative long-term health consequences, raising the possibility that stress is related to accelerated aging. In this study, we examine whether resilience factors affect stress-associated biological age acceleration. Recently developed “epigenetic clocks” such as GrimAge have shown utility in predicting biological age and mortality. Here, we assessed the impact of cumulative stress, stress physiology, and resilience on accelerated aging in a community sample (*N* = 444). Cumulative stress was associated with accelerated GrimAge (*P* = 0.0388) and stress-related physiologic measures of adrenal sensitivity (Cortisol/ACTH ratio) and insulin resistance (HOMA). After controlling for demographic and behavioral factors, HOMA correlated with accelerated GrimAge (*P* = 0.0186). Remarkably, psychological resilience factors of emotion regulation and self-control moderated these relationships. Emotion regulation moderated the association between stress and aging (*P* = 8.82e−4) such that with worse emotion regulation, there was greater stress-related age acceleration, while stronger emotion regulation prevented any significant effect of stress on GrimAge. Self-control moderated the relationship between stress and insulin resistance (*P* = 0.00732), with high self-control blunting this relationship. In the final model, in those with poor emotion regulation, cumulative stress continued to predict additional GrimAge Acceleration even while accounting for demographic, physiologic, and behavioral covariates. These results demonstrate that cumulative stress is associated with epigenetic aging in a healthy population, and these associations are modified by biobehavioral resilience factors.

## Introduction

Cumulative stress can have adverse psychiatric and physical effects, increasing risk for cardiometabolic diseases, mood disorders, post-traumatic stress disorder and addiction [1–11]. There are several potential psychological and biological mechanisms through which these effects may occur. For example, stress may reduce psychological resilience measures such as emotion regulation and self-control that are known to protect against psychiatric and physical health outcomes [1, 12–14]. Notably, emotional stress exposure decreases cognitive and emotion regulation abilities [15–18], and this effect may be modulated by cortisol [15]. Furthermore, stress decreases self-control abilities [19–21] and impacts the likelihood of individuals engaging in healthy behaviors such as exercise or maintaining a healthy diet, and is associated with unhealthy behaviors such as smoking, alcohol, and drug use [22–25]. Recent evidence also suggests that stress effects on metabolic health may be affected by BMI-related changes in insulin resistance and other gut hormones [26, 27]. Indeed, stress’s effects on physiology resulting in alterations in neuro-hormonal signaling pathways as well as increased inflammation are well documented [26, 28–30]. Both stress and these physiologic changes may increase the risk of multiple physical and psychiatric illnesses, which in turn increase morbidity and mortality risk. This has often been described as an increased allostatic load, and notably many of these processes, such as metabolic and cardiovascular dysfunction, have been associated with human aging [31]. For example, insulin signaling might be linked to aging and aging-related diseases in humans [32], with recent data on metformin (a treatment for insulin resistance) suggesting it may be useful as an anti-aging drug [33].

There is growing evidence that cumulative stress may adversely impact health via accelerating the cellular aging process. For example, stress shortens telomere length and alters telomerase activity, and this interaction is modified by behavioral and psychological resilience factors [34–37]. However, recent studies have demonstrated mixed results on whether characteristics that contribute to resilience improve or worsen the impact of stress on health [38–47]. These data suggest that resiliency factors may modulate the relationship between chronic stress and aging, but to our knowledge this has not been tested in a healthy community sample. While there are many aspects of resilience, including cultural/societal, environmental, and personal which can decrease the negative consequences of stressors on individuals, herein we will focus on personal-level, psychological skills, including self-control and emotion regulation.

Recently developed DNA methylation-based epigenetic “clocks” appear to provide a more accurate measure of biological age than telomere length [48–51]. These clocks are built from a set of DNA methylation markers that correlate with chronologic age and serve as molecular estimators of biological age in cells, tissues, and individuals [52]. Epigenetic clocks have a significantly higher predictive value than previously used measures such as telomere length for frailty, [53] mortality risk [54, 55], hazard ratios [56], and chronologic age [57]. The development of these biological aging markers promises to not only aid in identifying individuals at higher risk for aging-related illnesses, but potentially also developing interventions to prevent accelerated aging.

Previous studies (reviewed by Palma-Gudiel et al [58]) have utilized epigenetic clocks to demonstrate associations between trauma, early life adversity, or low socioeconomic status and accelerated epigenetic aging. Studies have often been focused upon selected populations, such military veterans [45], individuals with significant trauma histories [59], or specific cohorts at higher risk [60–62]. Notably, these studies did not exclude, and often explicitly included, individuals with significant mental and physical illnesses, including PTSD, MDD, and other disabilities [59, 63]. These studies also primarily utilized epigenetic clocks trained upon chronologic age. However, a recently developed epigenetic clock, GrimAge, was trained using biomarkers of mortality and indicators of health, and has superior performance in predicting health outcomes when compared with other epigenetic clocks [51, 64].

**Fig. 1:**
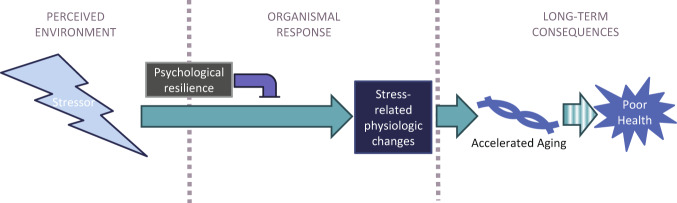
Model of relationships between cumulative stress, resilience, physiology, and aging. We hypothesize that stress is positively associated with accelerated biological aging, which we measure via GrimAge Acceleration (GAA), and that this relationship will be mediated by stress-related physiologic changes such as insulin and HPA signaling. We also hypothesize that strong psychological resilience factors will be protective against the negative consequences of stress on aging. Note that these relationships are predictive, not causative, as this study is cross-sectional and thus directionality of relationships cannot be conclusively examined.

We utilized GrimAge Acceleration (“GAA”, defined as the residual of the regression of GrimAge to chronologic age, with a positive number indicating biological age greater than chronologic age) to conduct a cross-sectional study to answer three questions. First, is cumulative stress related to epigenetic markers of biological aging in a healthy young-to-middle-aged community population? Second, if stress is associated with epigenetic aging, does stress-related physiology contribute to stress-associated biological aging? And finally, how do psychological factors that contribute to resilience modulate these relationships? Based on previous research, we hypothesized that cumulative stress will be positively associated with GrimAge Acceleration (GAA), that stress effects on GrimAge will be related to changes in the hypothalamic-pituitary-adrenal axis (HPA) and insulin sensitivity, and that strong emotion regulation as measured by the Difficulties in Emotion Regulation Scale (DERS, [65]) and high self-control as measured by the Self Control Scale-Brief (SCS-B, [66]) will moderate the relationships between stress, physiology, and accelerated aging (See Fig. 1 for a model summarizing our hypotheses).

## Materials and methods

### Cohort recruitment

The participant cohort included 444 community adults between the ages of 18–50 in the greater New Haven, CT area who volunteered to participate in a study examining the role of stress and self-control at the Yale Stress Center as previously described [67]. Briefly, participants were recruited via advertisements online, in local newspapers, and at a community center between 2008 and 2012. Participants were excluded if they had a substance use disorder (not including nicotine) as assessed via the Structured Clinical Interview for Diagnostic and Statistical Manual of Mental Disorders, 4th Edition (SCID-I for DSM-IVTR), were pregnant, had a chronic medical condition (e.g, hypertension, diabetes, hypothyroidism), or were unable to read English at or above the 6th grade level. Participants were also excluded if they had a concussion with loss of consciousness greater than 30 minutes, another head injury such as documented traumatic brain injury or another injury with documented lasting deficits, or were using any prescribed medications for any psychiatric or medical disorders. Breathalyzer and urine toxicology screens were conducted at each appointment to ensure the participants were drug-free. Of a total of 1000 potential participants who underwent initial screening for eligibility, epigenetic data combined with physiologic and behavioral data were available on 444, who comprised the current sample. All participants provided written and verbal informed consent to participate, and the research protocol was reviewed and approved by the Yale IRB.

### Initial assessment and measurement of physiologic parameters

All eligible subjects met with a research assistant for two intake sessions to complete a physical health review with the Cornell Medical Index (CMI, [68]), structured clinical interview for diagnoses (SCID) of DSM-IVTR psychiatric illnesses, cumulative stress interview, self-report assessments and a separate morning biochemical evaluation after fasting overnight. The structured clinical interview was performed by masters’ or doctoral level clinical research staff. Fasting insulin and glucose were obtained and Cortisol was assessed at four time-points, spaced 15 min apart beginning at 7:30 AM after overnight fasting and collected while participants were in a quiet and comfortable laboratory setting at the Yale Stress Center. Participants were financially compensated for participating in the study.

### Psychological measures

Cumulative stress was assessed using the Cumulative Adversity Inventory (CAI, [69]), a 140-item multifaceted interview-based assessment of life events and subjective stress through which trained interviewers asked participants about specific stressful events that occurred during their lifetime, which comprised the subscales of major life events, life trauma events and recent life events. For purposes of scoring, a “yes” to the specific stressful event occurring led to a “1” and a sum of all the “yes” endorsements comprised the subscale score for these events subscale. The final subscale of chronic stress was the participant’s own sense of feeling overwhelmed and unable to manage the events for the other subscales listed. This was rated on a “not true”, “somewhat true”, or “very true” scale, with assigned scores of 0, 1, and 2, respectively. The final score is a sum of these values for the chronic stress subscale. The CAI-total score was a sum of each of the subscale score with a higher score indicating a higher overall level of lifetime cumulative stress. The CAI has been demonstrated to have excellent overall reliability as reported in previous research [12, 26, 70–72]. In our population for this study, the alpha reliability is 0.86. It has been previously shown to predict cumulative stress related brain volume reductions and sensitized stress functional responses as well as prediction of physical, metabolic and behavioral responses [26, 70–72].

Emotion regulation was assessed using the Difficulties with Emotion Regulation Scale (DERS, [65]), which is a 41-item trait-level measure that assesses across domains of lack of emotional awareness, goals, clarity, strategies, acceptance, and impulse control in managing emotions. Higher scores on the DERS correspond to lower ability to regulate emotion. Alpha reliability has been reported to be >0.90 for the total score, and ≥0.80 for the sub-scores [65]. In this population, the alpha reliability is 0.92.

Self-control was assessed using the Self-Control Survey-Brief (SCS-B, [66]), which is a 13-item scale that assesses overall self-control. A higher score on the SCS-B suggests a stronger level of self-control. There are no sub-scores provided by the SCS-B, and the overall SCS-B has been reported to have an alpha reliability >0.80 [66]. The alpha reliability in this study is 0.85.

The Cornell Medical Index (CMI) was used to assess for participants’ current health. It is a 195-question interview that captures both physical and psychological health symptoms, and has been validated as an indicator for current general health in many studies [68, 73, 74]. A higher score on the CMI suggests more symptoms and worse overall health. The alpha reliability of the total CMI is 0.94. The psychological subscore has an alpha reliability of 0.92, and the biological subscore has a reliability of 0.90.

Cronbach alpha reliabilities for each of the scales described above were obtained using the alpha function in the R psych package [75].

### DNA methylation and epigenetic clock analysis

DNA for epigenetic analysis was collected from whole blood samples as previously described [67]. Briefly, all samples were profiled using Illumina Infinium HumanMethylation450 Beadchips, which covers 96% of CpG islands and 99% of RefSeq genes. Quality control on these data are as previously published [67]. They are described in brief below:

*Probe QC*: To ensure high-quality data, we set a more stringent threshold of *P* < 10^–12^. Intensity values showing *P* > 10^−12^ were set as zero. Additionally, we removed 11,648 probes on sex chromosomes and 36,535 probes within 10 base pairs of single-nucleotide polymorphisms. Finally, a total of 47,791 probes were removed and the remaining 437,722 probes were used for further analysis.

*Sample QC*: Using a detection *P* value < 10^–12^, one sample showing a call rate < 98% was excluded from analysis. Five samples showing sex discrepancy between the methylation predicted sex and self-reported sex were also excluded from analysis.

*Data processing and normalization*: Data processing and normalization were performed using the recently published protocol (Lehne et al., 2015). We first perform background correction and within-array normalization to the original green/red channel intensity data using the preprocessIllumina function in the minfi R package. The processed data were transformed to M/U methylation categories. Next, we separately performed between-array-normalization with the quantile method using the normalizeBetweenArrays function in the limma R package (version 3.26.2) after dividing the data matrix into 6 independent parts: Type I M Green, Type I M Red, Type I U Red, Type I U Green, Type II Red, Type II Green. The normalized data were merged and the beta value at each CpG site was determined.

After obtaining beta values, epigenetic clock analysis was performed as described in Lu et al. using the New Methylation Age Calculator at https://dnamage.genetics.ucla.edu/new [51]. Data were normalized as per their protocol, and the advanced analysis option was used. We focus on GrimAge acceleration (GAA), which is defined as the residuals of a linear correlation of GrimAge to chronologic age. No effects of array batch on GAA were observed (Supplementary Fig. 1).

The analyses herein were performed without accounting for individual variations in cell types. The Houseman method was used to determine cell type proportion [76], and the inclusion of cell fractions as covariates in a linear model does not impact the primary conclusions of this paper (see Supplementary material).

### Statistical analysis

Data organization and analysis were conducted using R 3.6.3 [77] and RStudio. Linear regressions were first implemented to examine univariate associations between independent and dependent variables. Multivariable linear regressions adjust for demographic (sex, race, years of education, marital status, income) and behavioral (smoking, alcohol use, and BMI) covariates unless otherwise stated. These covariates were selected due to prior work demonstrating a relationship to epigenetic aging. Chronologic age is incorporated into the model as part of the calculation of GAA (the residual of GrimAge regressed upon chronologic age). There was no significant correlation between chronologic age and GAA. Analyses of the relationship between CAI, GAA, psychological and physiologic variables were performed in both the univariate unadjusted model and the multivariate adjusted model accounting for demographic and behavioral measures, but except when the conclusions differ, statistical values in the text represent the multivariate models for simplicity. CAI, DERS, and SCS were mean-centered to address issues of collinearity (particularly regarding individual regression coefficients) when assessing for moderation.

All tests were two-tailed with alpha set at 0.05. Statistical significance in both standard linear regressions and moderation analyses were assessed from *t* values. *R*^2^ reported on plots represent the simple relationship between the stated variables, while adjusted R^2^ values in the text represent the model. Partial η^2^ values represent the effect size for the specific variable, with a value >= 0.01 typically indicating a small effect, >= 0.06 a medium effect, and >= 0.14 a large effect [78]. Wilcoxon signed-rank test was used to compare data between sexes. Mediation analysis was performed to determine if stress impacts GAA via behavioral and physiologic factors. Simple mediation effects were calculated via R using 10,000 simulations without bootstrapping using the mediation package [79]. Mediation was considered significant if the proportion mediated was greater than 0 with an alpha of 0.05. Serial mediation was calculated via R using the Lavaan package [71], with an indirect effect defined as the product of the coefficients of the effect of stress on BMI, of BMI on HOMA, and of HOMA on GAA. Assessment of the individual variables’ attributable GrimAge acceleration as well as confidence intervals were calculated using the Emmeans package using unadjusted pairwise comparisons.

## Results

### Demographics and clinical characteristics

As shown in Table 1, study participants were healthy and without evidence of medical or psychiatric diseases. The majority were non-smokers (79.6%), social drinkers with low risky alcohol intake screening scores (72.7% of participants have Alcohol Use Disorders Identification Test (AUDIT) < 8, and 91.7% < 15), and were not obese (74.5% of participants have a BMI < 30, 89.2% < 35). Both physical and psychological symptoms assessed on the Cornell Medical Index (CMI, [68]) were low, with 86% of participants scoring below the typical screening threshold of 30.

**Table 1:** Demographics of community population.

Category		Frequency^1^/mean^2^	5th% to 95th%	Stdev
Gender^1^	*Female*	55.2%		
	*Male*	44.8%		
Smoker^1^	*No*	79.6%		
	*Yes*	20.4%		
Race^1^	*White*	73%		
	*Black*	18%		
	*Other*	9%		
Marital Status^1^	*Never married*	73.4%		
	*Married*	16.2%		
	*Divorced/other*	10.4%		
Regular EtOH use^1^	*Yes*	70.4%		
	*No*	29.6%		
AUDIT^2^		5.94	0 – 19	5.97
BMI^2^		26.96	20–37.8	5.377
Days smoking past 4 weeks^2^		4.0	0–28	9.30
Days drinking past 4 weeks^2^		6.3	0–20	6.95
CAI-total score^2^		19.8	6–41	10.41
DERS^2^		69.9	43–108	19.73
Brief-SCS^2^		45.6	31–60	8.66
Age^2^		28.6	19–47	8.74
Years of Education^2^		15.4	12–20	2.47
Employment Income (monthly) ^2^		$1,010.59	0–$3500	$1,421.33
Cornell-biological subscore^2^		10.2	1–30	9.1
Cornell-psychological subscore^2^		5.3	0–20	6.7
Cornell-total^2^		15.5	2–46	14.48
Cortisol/ACTH (AUC) ^2^		0.30384	0.0988–0.741	0.21344
HOMA^2^		3.169	1.05 – 7.13	1.9697

^1^Frequency

^2^Mean

Demographics and average statistics for the test population

*AUDIT* alcohol use disorder identification test, *BMI* body mass index, *CAI* cumulative adversity index, *DERS* difficulty with emotion regulation scale, *SCS* self-control scale, *HOMA* homeostatic model assessment of insulin resistance

### Cumulative stress predicts accelerated biological aging as measured by GrimAge

**Fig. 2:**
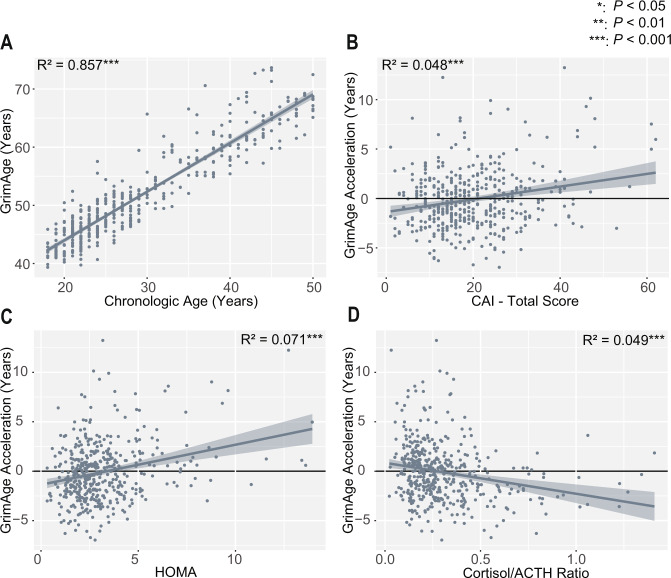
GrimAge and GrimAge acceleration correlate with cumulative stress and physiologic stress pathways. **A** Chronologic age significantly predicts GrimAge (*P* < 2e−16). **B** Cumulative stress total as measured by the CAI (CAI-Total) significantly predicts GAA before (*P* = 2.00e−6) and after accounting for covariates. **C** Higher insulin resistance (as measured by HOMA) shows a significant positive correlation with GAA before (*P* = 1.11e−8) and after accounting for covariates. **D** The Cortisol/ACTH ratio is negatively correlated with GAA before accounting for covariates (*P* = 2.39e−6), but not afterward. *P* and *R*^2^ values in the figure represent simple univariate models (Y ~ X). In the main text, models are adjusted for covariates as stated.

As expected, there was a high association between individuals’ chronologic age and GrimAge (Age: *t* = 51.4, *P* < 2e−16, adjusted *R*^2^ = 0.856, Fig. 2A). This relationship is not altered by inclusion of the covariates of smoking, alcohol use, BMI, race, sex, income, and years of education (Age: *t* = 49.1, *P* < 2e−16, partial η^2^ = 0.848; model (GrimAge ~ Age + covariates) adjusted *R*^2^ = 0.912), and this relationship remained significant accounting for cellular fractions (Supplementary Table 1). Also, using a univariate linear regression, greater cumulative stress as measured by the total Cumulative Adversity Index (CAI) score significantly predicted higher GAA (CAI: *t* = 4.82 *P* = 2.00e−6, η^2^ = 0.050, adjusted *R*^2^ = 0.0478, Fig. 2B). While there were significant differences in GAA based on sex (*P* = 1.33e−7), both males (CAI: *P* = 3.35e−4, adjusted *R*^2^ = 0.0586) and females (CAI: *P* = 3.12e−5, adjusted *R*^2^ = 0.0652) demonstrated similar correlations between stress and GAA. Further analysis showed these results are consistent across CAI subscales, as well as with the Childhood Trauma Questionnaire and several of its subscales (Supplementary Table 2).

After accounting for the covariates of smoking, alcohol use, BMI, race, sex, income, and years of education, the relationship between GAA and CAI remains significant (CAI: *t* = 2.073, *P* = 0.0388, partial η^2^ = 0.010; model (GAA ~ CAI-total + covariates): adjusted *R*^2^ = 0.3869); individual covariate effects shown in Supplementary Table 3). When considered as potential mediators of the relationship between stress and GAA, BMI (proportion mediated = 0.288, *P* = 0.0042) and smoking (proportion mediated = 0.443, *P* = 0.0030), but not alcohol use (proportion mediated = 0.001, *P* = 0.931), show partial mediating effects (Supplementary Table 4).

Consistent with the underlying assumption that GAA is related to measures of health, GAA also predicted psychological and physical health symptoms as measured by the CMI (Supplementary Fig. 2A; total CMI: *t* = 3.449, *P* = 6.18e−4, adjusted *R*^2^ = 0.024).

### Stress-related physiology is associated with GrimAge acceleration

Given the known relationship between cumulative stress and physiology, we assessed the relationship between the stress-related physiologic factors of insulin resistance and HPA-axis signaling and GAA. We found that higher HOMA (a measure of insulin resistance) significantly predicted GAA (Fig. 2C, HOMA: *t* = 2.362, *P* = 0.0186, partial η^2^ = 0.013; model (GAA ~ HOMA + Covariates): adjusted *R*^2^ = 0.389).

We then assessed whether cortisol/ACTH ratio changes impacted GAA. Indeed, low cortisol/ACTH ratio, a measure of adrenal sensitivity, was associated with GAA in a simple univariate model, (Fig. 2D, Cort/ACTH ratio: *t* = −4.78, *P* = 2.39e−6, η^2^ = 0.049, adjusted *R*^2^ = 0.0470), though this becomes non-significant when accounting for covariates (Cort/ACTH ratio: *t* = −0.721, *P* = 0.471, partial η^2^ = 0.001; model (GAA ~ Cort/ACTH + Covariates): adjusted *R*^2^ = 0.3816). We also find a significant association between stress and Cortisol/ACTH ratio (Supplementary Fig. 2B, CAI: *t* = −2.146 *P* = 0.0324; model (Cort/ACTH ratio ~ CAI + covariates): adjusted *R*^2^ = 0.2197).

### Emotion regulation moderates the relationship between stress and GrimAge acceleration directly

**Fig. 3:**
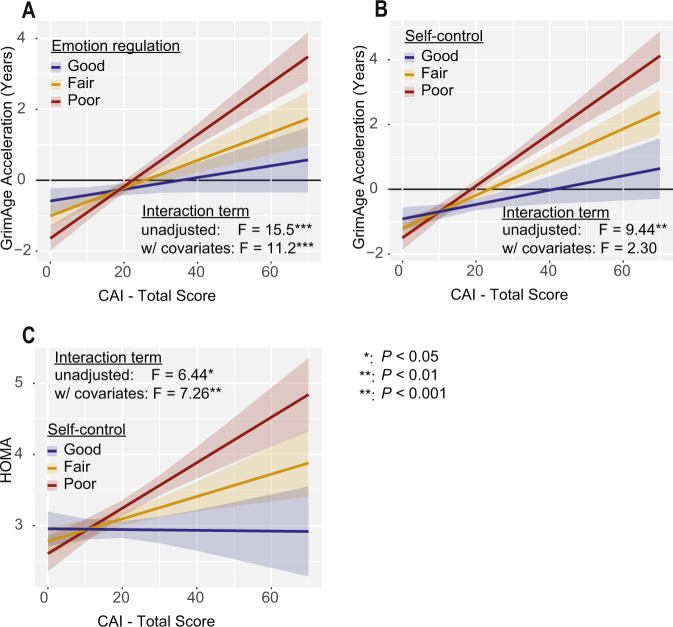
Psychological resilience factors moderate the effects of cumulative stress on GrimAge Acceleration and physiologic stress pathways. **A** Individuals with stronger emotion regulation (as measured by lower DERS scores) suffer less GAA at high stress than individuals with poor emotion regulation before (GAA ~ CAI X DERS *P* = 9.51e−5; GAA ~ CAI X DERS + Covariates: *P* = 8.82e−4) and after accounting for covariates. For panel A, “good” represents the slope at the 25th percentile of DERS, “fair” at the 50th percentile, and “poor” the 75th percentile. **B** Better self-control (as measured by higher B-SCS scores) is protective against the effects of stress on GAA before accounting for covariates (GAA ~ CAI X SCS *P* = 0.00226; GAA ~ CAI X SCS + Covariates: *P* = 0.130), but not after including them in the model. **C** Stronger self-control moderates the relationship between stress and insulin resistance before (HOMA ~ CAI X SCS *P* = 0.0115; HOMA ~ CAI X SCS + Covariates *P* = 0.00732) and after accounting for covariates. For panels (B) and (C), “good” represents the slope at the 75th percentile of B-SCS, “fair” at the 50th percentile, and “poor” the 25th percentile.

We then asked whether the relationship between cumulative stress and epigenetic aging was modulated by characteristics that contribute to an individual’s psychological resilience. We hypothesized that strong emotion regulation abilities would be protective against stress-related accelerated aging. We found that emotion regulation as assessed by the Difficulties in Emotion Regulation Scale (DERS, [65]) significantly moderated the relationship between GAA and CAI (Fig. 3A, CAI:DERS: *F* = 11.22, *P* = 8.82e−4, partial η^2^ = 0.025; model (GAA ~ CAI X DERS + covariates): adjusted *R*^2^ = 0.4004), such that poor emotion regulation significantly increased the effects of CAI on GAA. There was not a significant difference between males and females in emotion regulation (*P* = 0.0949).

### Self-control moderates the association between stress and insulin resistance, which is associated with GrimAge acceleration

We next assessed whether psychological resilience in the form of self-control (as measured via the SCS-B, [66]) alters the association between cumulative stress and GAA. We found higher self-control is protective against the effects of stress on GAA before accounting for covariates, but the interaction became non-significant when covariates were accounted for (Fig. 3B, CAI:SCS: *F* = 2.303, *P* = 0.130, partial η^2^ = 0.005; model (GAA ~ CAI X SCS + Covariates: adjusted *R*^2^ = 0.3874).

Given the potential interplay between self-control, insulin resistance, and stress, we next asked whether self-control moderated the relationship between stress and HOMA. We observed that, even when covariates are accounted for, self-control moderates the positive relationship between stress and HOMA, with stronger self-control blunting their relationship (Fig. 3C, CAI:SCS: F = 7.263, *P* = 0.00732, partial η^2^ = 0.017; model (HOMA ~ CAI X SCS + Covariates: adjusted *R*^2^ = 0.2871). Notably, self-control does not moderate the relationship between CAI and BMI (CAI:SCS: *F* = 0.679, *P* = 0.41). Self-control did not significantly differ between males and females (*P* = 0.0550).

### Exploratory mediation analyses suggest stress influences GrimAge via BMI and HOMA

While our ability to draw causative inferences are limited by the cross-sectional nature of our data, we used mediation analyses to explore potential relationships between weight, insulin resistance, and GAA. We hypothesized that the effects of BMI on GAA may be mediated through insulin resistance. Indeed, mediation analysis suggested that a significant portion of the effect of BMI on GAA may be mediated through HOMA (Supplementary Fig. 3A, proportion mediated = 0.247, *P* = 0.02). Given these findings, we next asked whether BMI and insulin resistance act sequentially to mediate the effects of stress on GAA. We identified a significant indirect effect, suggesting that stress may affect GAA through increased BMI and elevated insulin resistance (Supplementary Fig. 3B, indirect effect = 0.003; *P* = 0.030), though there continues to be a significant direct effect of stress on GAA as well (direct effect = 0.034, *P* = 0.009).

### Cumulative stress and estimated change in GrimAge

Finally, we sought to identify the comparative contributions of our significant variables to GAA. To do this, we constructed a linear regression model using all demographic covariates (sex, race, marital status, education, income), behavioral covariates (smoking, alcohol, BMI), physiologic factors (HOMA, Cortisol/ACTH ratio), and psychological factors. In this model, we continue to see a significant interaction between stress and emotion regulation in relation to GAA (CAI:DERS *t* = 3.424, *P* = 0.000677, partial η^2^ = 0.027; model (GAA ~ CAI-total X DERS + HOMA + Cort/ACTH ratio + SCS + Covariates): adjusted *R*^2^ = 0.4056). Notably in this model, HOMA (*t* = 2.308, *P* = 0.0215, partial η^2^ = 0.012), BMI (*t* = 2.641, *P* = 0.00857, partial η^2^ = 0.016), and smoking (*t* = 10.47, *P* < 2e−16, partial η^2^ = 0.204) also demonstrate significant effects on GAA. The impact of the cortisol/ACTH ratio on GAA is not significant (*t* = −0.668, *P* = 0.504, partial η^2^ = 0.001), and its removal from the model does not impact any of the above conclusions.

Using this final linear model, we estimated the changes in GrimAge for each significant variable (Table 2) using estimated marginal means [80]. When comparing the effects of high stress (CAI-total: 75th percentile) versus low stress (CAI-total: 25th percentile) in those with poor emotion regulation (DERS: 75th percentile), stress was associated with half a year of aging independent of all other covariates and physiologic factors. However, when emotion regulation was strong (DERS: 25th percentile), stress did not independently predict GAA. Again comparing 75th versus 25th percentiles, BMI independently was related to an increase of 0.46 years of GrimAge, and HOMA for ¼ of a year. We also identified daily smoking (3.8 years), male sex (1.2 years), self-identifying as Black (1 year), and never having married (0.71 years) as covariates that significantly predicted accelerated GrimAge. When accounting for cellular fractions we see similar results regarding the relationships between stress, emotion regulation, and GAA. However, when accounting for cellular fractions, the associations between GAA and both HOMA and marital status become non-significant (Supplementary Table 5). Prior literature [51] suggests that GrimAge predicts the hazard ratio exponentially (HR = 1.1^GAA^). Thus, each additional year of GAA would be expected to increase the relative risk of death by approximately 10%.

**Table 2:** Estimated change in GrimAge with significant variables in final model.

Independent variables	Comparison	Attributable GrimAge acceleration (Years)	Conf int (5% - 95%)	*p* value
Stress (poor emotion reg.)	CAI: 25th% vs 75th%; DERS at 75th%	0.48	0.164 to 0.794	0.003
Stress (good emotion reg.)	CAI: 25th% vs 75th%; DERS at 25th%	−0.04	−0.426 to 0.341	0.8276
HOMA	25th% vs 75th%	0.27	0.041 to 0.506	0.0215
BMI	25th% vs 75th%	0.46	0.118 to 0.803	0.0086
Smoking	none vs daily	3.79	3.08 to 4.5	<0.0001
Race	White vs Black	1.04	0.421 to 1.655	0.001
Sex	Female vs male	1.2	0.726 to 1.68	<0.0001
Marital status	Married vs never married	0.71	0.073 to 1.345	0.0291

Analysis of estimated change in GrimAge (dependent variable) for different independent variables in the final model. Comparisons for continuous variables are made between 25th percentile and 75th percentile, with a positive value signifying higher GrimAge in the 75th percentile. Categorical variables are compared as listed, with a positive value signifying higher GrimAge in the 2nd listed group.

*BMI* body mass index, *CAI* cumulative adversity index, *DERS* difficulty with emotion regulation scale, *HOMA* homeostatic model assessment of insulin resistance

## Discussion

In this study, we report novel findings that cumulative stress is associated with accelerated epigenetic aging in a healthy, young-to-middle-aged community sample, even after adjusting for sex, race, BMI, smoking, alcohol use, income, marital status, and education. Epigenetic aging was measured by GrimAge, a marker which has previously been associated with increased morbidity and mortality and correlates with physical and psychological health symptoms in our study. The relationship between stress and age acceleration is most prominent in those with poor emotion regulation and was related to behavioral factors such as smoking and BMI. Both stress and GAA were associated with changes in insulin resistance, which was moderated via self-control. These results suggest a relationship between stress, physiology, and accelerated aging that is moderated by emotion regulation and self-control. Overall, these findings point to multiple potentially modifiable biobehavioral targets of intervention that may reduce or prevent the deleterious effects of stress on aging and long-term health outcomes.

This study included a generally healthy, young-to-middle-aged community population, yet we still identified a significant relationship between cumulative stress and age acceleration. The population was taking no prescription medications for any medical conditions, nor were they suffering from current mental illnesses, including major depressive disorder or generalized anxiety disorder. The study includes individuals with obesity, as well as a small number of individuals with risky drinking levels as determined by the AUDIT scores. The frequency of these individuals in the sample is generally in line with those in a community population, and thus we included alcohol use and BMI as covariates to account for the impact of these variables on the results. Prior work has demonstrated that GrimAge better predicts mortality than other epigenetic clocks, and GrimAge predicts lifespan more accurately than self-reporting smoking history, demonstrating that GrimAge is a biologically meaningful and potentially clinically useful biomarker for health [51, 64]. Our findings are consistent with recent work showing that those with significant trauma histories [59, 81] or with diagnoses of mental illnesses, such as Bipolar disorder or MDD, may experience accelerated aging as measured by epigenetic clocks [57, 81–84]. In particular, this study builds on previous findings by Zannas et al that demonstrated a relationship between trauma and epigenetic aging using the Horvath clock. However, to the best of our knowledge this is the first study to investigate the impact of cumulative stress on epigenetic aging in a healthy community sample without significant physical or mental illness. Also it is the first to our knowledge to identify factors that contribute to psychological resilience as potential modulators of such an effect. This opens the possibility that the distinction between the effects of stress on pathologic and non-pathologic samples may be along a continuum. It would be interesting to examine resilience characteristics in the population studied by Zannas et al to determine if there is a limit to the protective effects of psychological resilience. Thus, preventive interventions that decrease stress and improve resilience may be useful for maintaining long-term mental and physical health.

The relationship between stress and epigenetic aging appears to be modulated via specific psychological traits, including emotion regulation and self-control. Those with better emotion regulation and higher levels of self-control were observed to have less age acceleration even at similar levels of stress. Indeed, based on their GAA, our estimates indicate that the relationship between stress and GrimAge is as powerful as BMI, but only for those with poor emotion regulation. As these are skills that may be developed through specific psychological interventions [85], these results raise the possibility that building emotion regulation skills could result in improvements in epigenetic aging, morbidity, and mortality [86] for these populations. As this is a cross-sectional study, we are not able to address whether these relationships are causal. These novel cross-sectional findings provide support for potential future research that may assess whether such an intervention could positively impact epigenetic aging and other indices of long-term health outcomes. Other studies could also examine different aspects of resilience, such as cultural or environmental factors that contribute to resilience to determine if they also are protective against the effects of stress on epigenetic age acceleration. Future studies could also explore other physiologic mechanisms through which psychological resilience may influence epigenetic aging. Based on prior work, inflammation could be particularly important for this relationship. In particular, prior studies have found C-reactive protein [87] and IL-6 [88] to be related to emotion regulation and measures of health. The work by Gianaros et al suggests that neurologic activity of the dorsal anterior cingulate cortex may be involved as well.

The relationship between cumulative stress, epigenetic aging, and insulin resistance is of particular note given the prominence of insulin signaling in aging-related pathways [89, 90], as well as current trials investigating metformin as a potential anti-aging drug [33]. In association with this body of work, our study suggests insulin resistance as at least one factor through which stress is associated with accelerated aging, even in a healthy population not suffering from diabetes. As this study is limited by its cross-sectional nature, any causal hypotheses regarding interactions between stress, BMI, insulin resistance, and aging will require longitudinal data to draw specific inferences beyond correlative relationships. Longitudinal studies would also enable prospective assessments of stress, which may be less subject to recall bias based on their current context. This study also identifies the cortisol/ACTH ratio as a potential point of connection between stress and epigenetic aging. However, this measure is somewhat limited in that it reflects an acute measure of the HPA axis, and this relationship becomes non-significant with the inclusion of our covariates. Future studies could utilize other, longer-term measures of HPA axis function such as hair cortisol to better characterize the relationship between stress, epigenetic aging, and the HPA axis.

Nonetheless, this study is the first to identify a clear relationship between cumulative stress and GrimAge acceleration in a healthy population, which suggests stress may play a role in accelerated aging even prior to the onset of chronic diseases. Notably, this relationship was strongly moderated by resilience factors, including self-control and emotion regulation. We also identified smoking, BMI, insulin signaling, and potentially HPA signaling as mediators of this response. However, even when accounting for all these factors as well as demographic covariates such as race, cumulative stress continues to demonstrate a significant impact on GAA, suggesting other mechanisms relating stress to aging not identified herein are also present.

## Supplementary information

Supplementary Material

Supplementary Figure 1

Supplementary Figure 2

Supplementary Figure 3

## Data Availability

R scripts utilized for data analysis are available by contacting the authors directly.
