# Supplementary material for: Psychological and biological resilience modulates the effects of stress on epigenetic aging
Source: Transl Psychiatry. 2021 Nov 27;11:601. doi: 10.1038/s41398-021-01735-7 (PMC8627511; doi:10.1038/s41398-021-01735-7)
Supplement: Supplementary file 1 — Supplementary Material [file 41398_2021_1735_MOESM1_ESM.docx]

# Supplementary Material

As a secondary analysis, we accounted for cell count proportions by utiliing the Houseman method was to determine cell type proportion(Houseman et al., 2012). When we incorporate the cellular fractions into our linear models, we do not observe significant changes to our primary conclusions. The below Tables (Supplementary Tables 1 and 5) summarize the results.

### Supplementary Table 1: Accounting for cellular fractions does alter the primary conclusions of this article

When we re-assess our hypothesis accounting for cellular fractions via incorporating them into the linear model as covariates, we do not see significant alterations in the primary conclusions to our paper. The one result observed where there is discordance is when assessing whether there is an interaction between self-control and cumulative adversity on GrimAge Acceleration (bolded below). When we account for cellular fractions in addition to our standard covariates, we observe a significant moderating effect of self-control on the relationship between CAI and GAA. However, when cellular fractions are not accounted for, this moderating effect is not significant.

| Model | Without cell fractions | | With cell fractions | |
| --- | --- | --- | --- | --- |
|  | Coefficient | P value | Coefficient | P value |
| GrimAge ~ Age | Age: 0.836 | < 2e-16 | Age: 0.826 | < 2e-16 |
| GrimAge ~ Age + Covariates | Age: 0.823 | < 2e-16 | Age: 0.812 | < 2e-16 |
| GAA ~ CAI | CAI: 0.0642 | 2.00e-6 | CAI: 0.0655 | 3.01e-7 |
| GAA ~ CAI (Males) | CAI: 0.0785 | 3.35e-4 | CAI: 0.0883 | 2.92e-5 |
| GAA ~ CAI (Females) | CAI: 0.0690 | 3.12e-5 | CAI: 0.0671 | 2.31e-5 |
| GAA ~ CAI + Covariates | CAI: 0.00242 | 0.0388 | CAI: 0.0028 | 0.00985 |
| GAA ~ HOMA + Covariates | HOMA: 0.158 | 0.0186 | HOMA: 0.125 | 0.0468 |
| GAA ~ Cort/ACTH | Cort/ACTH: -3.13 | 2.39e-6 | Cort/ACTH: -2.63 | 6.26e-5 |
| GAA ~ Cort/ACTH + Covariates | Cort/ACTH: -0.434 | 0.471 | Cort/ACTH: -0.502 | 0.370 |
| GAA ~ CAI X DERS + Covariates | CAI:DERS: 0.00158 | 8.82e-4 | CAI:DERS: 0.00148 | 8.50e-4 |
| GAA ~ CAI X SCS + Covariates | **CAI:SCS: -0.00200** | **0.130** | **CAI:SCS: -0.00246** | **0.0436** |

### Supplementary Table 2: GrimAge Acceleration is strongly correlated to multiple measures of cumulative stress

GAA demonstrates significant correlations with multiple scores and subscores measuring stress, trauma, and other negative life events. Table shows the p values and adjusted R^2^ values for the univariate linear regression of GAA with a given measure.

| Stress Measure | *P* value | Adjusted R^2^ |
| --- | --- | --- |
| CAI - total | 2.00E-06 | 0.0478 |
| Chronic Stress Subscore | 6.79E-03 | 0.0148 |
| CAI without chronic stress | 2.30E-07 | 0.0589 |
| Major Life Events Subscore | 2.61E-03 | 0.01881 |
| Traumatic Life Events Subscore | 2.78E-07 | 0.05808 |
| Recent Life Events Subscore | 2.18E-03 | 0.01958 |
| CTQ score | 5.04E-04 | 0.02582 |
| Minimization/Denial Subscore | 0.606 | -0.001724 |
| Emotional Abuse Subscore | 0.0198 | 0.01038 |
| Physical Abuse Subscore | 4.12E-06 | 0.0465 |
| Sexual Abuse Subscore | 6.77E-05 | 0.03444 |
| Emotional Neglect Subscore | 0.14 | 0.00278 |
| Physical Neglect Subscore | 0.103 | 0.003893 |

### Supplementary Table 3: No individual covariate accounts for the effect of stress on GrimAge acceleration

Cumulative stress continues to significantly impact GAA when accounting for each covariate individually.

| Covariate: | *P* value of CAI for model: GAA~CAI + listed covariate | *P* value for covariate |
| --- | --- | --- |
| Sex | 3.92E-08 | 1.83E-08 |
| Race | 1.18E-04 | 6.12E-04 |
| BMI | 5.08E-04 | 5.78E-08 |
| Marital status | 8.84E-07 | 0.0131 |
| Age | 1.11E-06 | 0.263 |
| Income | 1.23E-06 | 0.0132 |
| Smoking | 1.31E-03 | <2E-16 |
| Years Education | 6.16E-05 | 1.39E-05 |
| Alcohol use | 1.13E-06 | 0.10 |

### Supplementary Table 4: BMI and smoking, but not alcohol use, partially mediate the effects of stress on GrimAge Acceleration

Mediation analyses demonstrate that BMI and smoking partially (but not fully) mediate the effects of stress (independent variable) on GAA (dependent variable), even when accounting for other covariates. Beta estimates in table reflect model accounting for all other covariates (including other behavioral covariates). * p ≤ 0.05, ** p ≤ 0.01, *** p ≤ 0.001.

| Mediator: | BMI | Smoking | Alcohol use |
| --- | --- | --- | --- |
| Direct effect of stress on mediator | 0.0987*** | 0.136** | 0.0035 |
| Direct effect of mediator on GAA | 0.0994*** | 0.142** | 0.0208 |
| Direct effect of stress on GAA | 0.0241* | 0.0241* | 0.0242* |
| Indirect effect of stress on GAA | 0.00985*** | 0.0193** | 7.01e-5 |

### Supplementary Table 5: When accounting for cellular fractions, stress continues to predict GAA specifically in individuals with poor emotion regulation

When we reassess our final model using estimated marginal means while accounting for cellular fractions, we continue to see a differential effect of stress dependent on emotion regulation. When accounting for cellular fractions, the effects of HOMA on GAA are no longer statistically significant, nor are the effects of marital status.

| Independent Variables | Comparison | Attributable GrimAge Acceleration (Years) | Conf int (5% - 95%) | P value |
| --- | --- | --- | --- | --- |
| Stress  (poor emotion reg.) | CAI: 25^th^% vs 75^th^%; DERS at 75^th^% | 0.49 | 0.200 to 0.787 | 0.0010 |
| Stress  (good emotion reg.) | CAI: 25^th^% vs 75^th^%; DERS at 25^th^% | -0.00042 | -.356 to 0.356 | 0.998 |
| HOMA | 25^th^% vs 75^th^% | 0.204 | -0.0143 to 0.423 | 0.0669 |
| BMI | 25^th^% vs 75^th^% | 0.33 | 0.0112 to 0.646 | 0.0425 |
| Smoking | none vs daily | 3.74 | 3.07 to 4.4 | <0.0001 |
| Race | White vs Black | 1.38 | 0.658 to 2.114 | <0.0001 |
| Sex | Female vs male | 1.58 | 1.04 to 2.12 | <0.0001 |
| Marital status | Married vs never married | 0.61 | -0.0951 to 1.323 | 0.105 |

### Supplementary Figure 1: There is no relationship between array batch and GrimAge Acceleration

When we assess for potential batch effects of arrays on GAA, we see no arrays where the average GAA differs significantly from 0 (all *P*_adjust_ > 0.05). Bonferroni correction was used to adjust for multiple comparisons (# of tests = 43). Points represent mean, and error bars indicate standard deviation.

### Supplementary Figure 2: GAA and stress correlate with markers of health and physiology

(A) The total Cornell Medical Index (Grey line, *P* = 6.18e-4) show positive correlation with GAA. Correlations for the Biological (Blue line, *P* = 9.50e-4) and Psychological (Red line, *P* = 0.00378) subscales are also significant. (B) The cortisol/ACTH ratio is negatively correlated with cumulative stress (*P* = 1.75e-4). *P* and R^2^ values in figure represent simple univariate models (Y ~ X). Statistics accounting for covariates are provided in the main text where appropriate.

### Supplementary Figure 3: BMI and HOMA partially mediate the relationship between stress and GrimAge Acceleration

(A) The effects of BMI on GAA are partially mediated through Insulin resistance (proportion mediated = 0.247, *P* = 0.02). (B) The effects of stress on GAA are serially mediated by BMI and HOMA (indirect effect = 0.003; *P* = 0.030). For the mediation models, coefficients of the relationships in the multivariate adjusted model are listed in the figure. The following symbols are used: * p ≤ 0.05, ** p ≤ 0.01, *** p ≤ 0.001.

# References

Houseman, E. A., Accomando, W. P., Koestler, D. C., Christensen, B. C., Marsit, C. J., Nelson, H. H., . . . Kelsey, K. T. (2012). DNA methylation arrays as surrogate measures of cell mixture distribution. *BMC Bioinformatics, 13*(1), 86. doi: 10.1186/1471-2105-13-86
