# Supplementary figures and images for: Psychological and biological resilience modulates the effects of stress on epigenetic aging
Source: Transl Psychiatry. 2021 Nov 27;11:601. doi: 10.1038/s41398-021-01735-7 (PMC8627511; doi:10.1038/s41398-021-01735-7)

Supplementary Figure 1

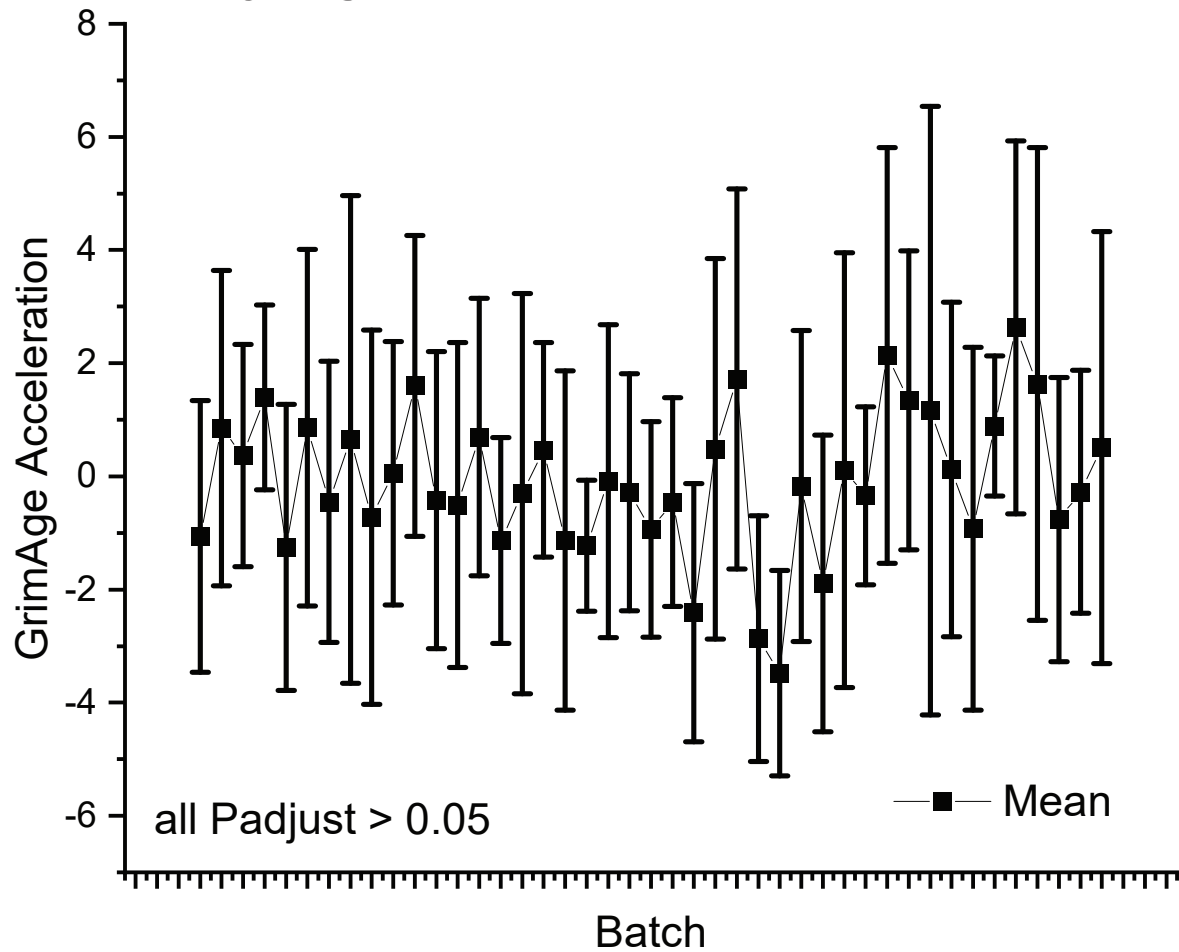

Supplement: Supplementary file 2 — Supplementary Figure 1 [file 41398_2021_1735_MOESM2_ESM.pdf]

# Supplementary Figure 2

A

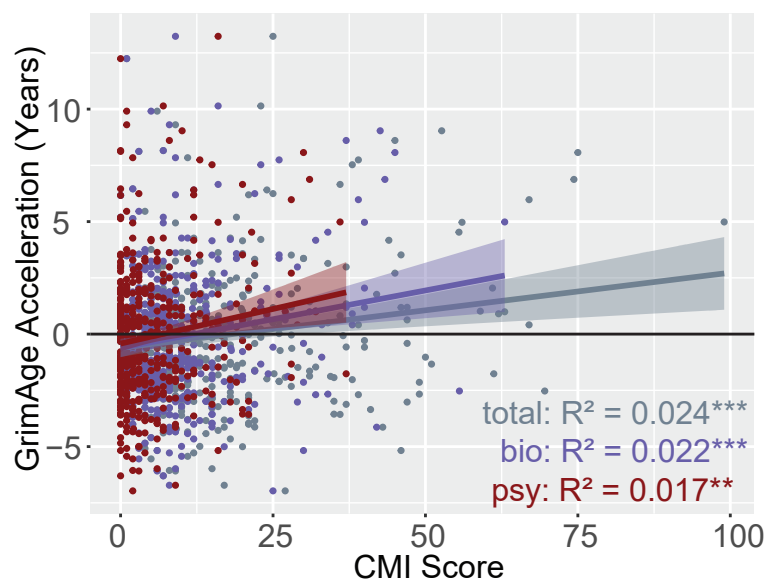

B

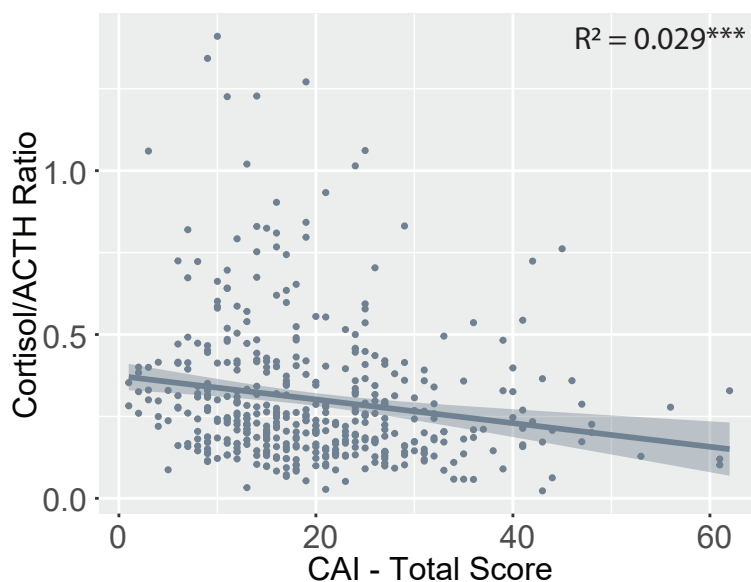

\*:  $P < 0.05$   
\*\*:  $P < 0.01$   
\*\*\*:  $P < 0.001$

Supplement: Supplementary file 3 — Supplementary Figure 2 [file 41398_2021_1735_MOESM3_ESM.pdf]

# Supplementary Figure 3

**A**

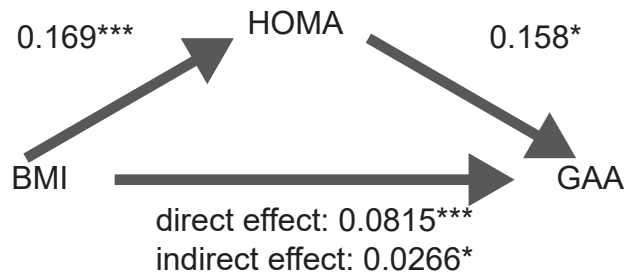

**B**

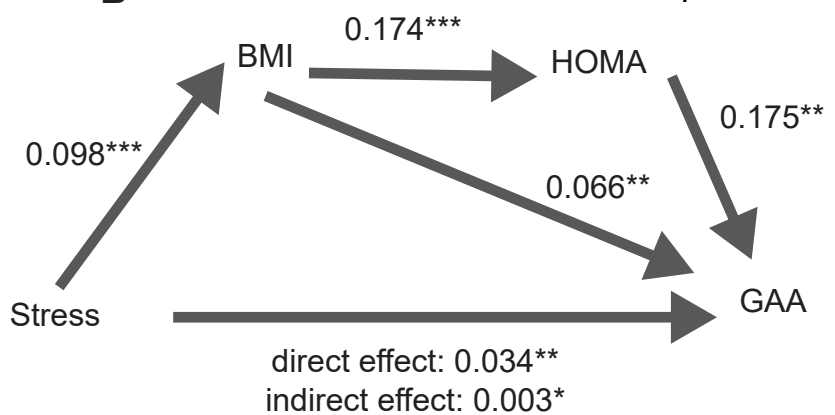

\*:  $P < 0.05$   
\*\*:  $P < 0.01$   
\*\*\*:  $P < 0.001$

Supplement: Supplementary file 4 — Supplementary Figure 3 [file 41398_2021_1735_MOESM4_ESM.pdf]
